# Contrasting maternal and paternal genetic histories among five ethnic groups from Khyber Pakhtunkhwa, Pakistan

**DOI:** 10.1038/s41598-022-05076-3

**Published:** 2022-01-19

**Authors:** Muhammad Tariq, Habib Ahmad, Brian E. Hemphill, Umar Farooq, Theodore G. Schurr

**Affiliations:** 1 Centre for Omic Sciences, Islamia College, Peshawar, 25120 Khyber Pakhtunkhwa Pakistan; 2 Department of Genetics, Hazara University Mansehra, Mansehra, 21120 Pakistan; 3 Department of Anthropology, University of Alaska, Fairbanks, AK 99775 USA; 4 College of Life Science, Northeast Forestry University, Harbin, 150040 China; 5 Department of Anthropology, University of Pennsylvania, 3260 South Street, Philadelphia, PA 19104-6398 USA

**Keywords:** Evolution, Genetics

## Abstract

Northwest Pakistan has served as a point of entry to South Asia for different populations since ancient times. However, relatively little is known about the population genetic history of the people residing within this region. To better understand human dispersal in the region within the broader history of the subcontinent, we analyzed mtDNA diversity in 659 and Y-chromosome diversity in 678 individuals, respectively, from five ethnic groups (Gujars, Jadoons, Syeds, Tanolis and Yousafzais), from Swabi and Buner Districts, Khyber Pakhtunkhwa Province, Pakistan. The mtDNAs of all individuals were subject to control region sequencing and SNP genotyping, while Y-chromosomes were analyzed using 54 SNPs and 19 STR loci. The majority of the mtDNAs belonged to West Eurasian haplogroups, with the rest belonging to either South or East Asian lineages. Four of the five Pakistani populations (Gujars, Jadoons, Syeds, Yousafzais) possessed strong maternal genetic affinities with other Pakistani and Central Asian populations, whereas one (Tanolis) did not. Four haplogroups (R1a, R1b, O3, L) among the 11 Y-chromosome lineages observed among these five ethnic groups contributed substantially to their paternal genetic makeup. Gujars, Syeds and Yousafzais showed strong paternal genetic affinities with other Pakistani and Central Asian populations, whereas Jadoons and Tanolis had close affinities with Turkmen populations from Central Asia and ethnic groups from northeast India. We evaluate these genetic data in the context of historical and archeological evidence to test different hypotheses concerning their origins and biological relationships.

## Introduction

South Asia is thought to be one of the first major geographic regions to be inhabited by anatomically modern humans (AMHs) as they dispersed out of Africa^1–4^. Archeological and anthropological evidence suggests the initial settlement of the region by AMH populations occurred 60–70 thousand years ago (kya)^3–13^, presumably via a coastal route^1,9,14–16^. From this region, modern humans likely dispersed into East Asia, Southeast Asia, and Sahul^2,4,17^. Although Paleolithic and Mesolithic peoples left their mark in the area^10^, major prehistoric and historic events with possible genetic consequences also occurred during the Neolithic period and later^18,19^.

Today, South Asia is home to more than one billion people who belong to thousands of distinct socio-culturally, ethnically, and genetically diverse populations^17,20–27^. These include more than 2200 major population groups^28^, over 450 tribal communities^29^ and some 30 hunter-gatherer populations^30,31^. Ethnic groups claiming to have arrived and settled in South Asia include but are not limited to Afghans, Arabs, Armenians, Aryans, Chinese, Greeks, Huns, Iranians, Mongols, Persians, Scythians, Syrians, Tajiks, Turks, and Uzbeks^32^. The overwhelming majority of the current ethnic groups are reportedly endogamous^31,33^, and speak an array of languages from various language families, including Indo-European, Dravidian, Tibeto-Burman, Austro-Asiatic, and Sino-Tibetan, each being differentially distributed throughout South Asia^34–36^.

This differential distribution has been attributed to the impact of external influences on South Asian populations. The earliest evidence of farming-based economies in South Asia has been traced to the introduction of West Asian cultigens such as wheat and barley at Mehrgarh, Pakistan, dating to 8 kya^37–41^. From there, farming and sedentary lifeways spread further east, laying the foundation for the later Indus Valley (including the cities of Harappa and Mohenjo-Daro) and the Gangetic Valley civilizations, which arose between 4.6–3.9 and 3.5–2.5 kya, respectively^40,41^. Sometime around 3.5 kya, Indo-European-speaking nomadic pastoralists from the southern steppes, often called ‘Aryans’, crossed the Hindu Kush Mountains and expanded into the subcontinent^42–46^. Later, in the eighth century CE, an Arab-Muslim army invaded Sindh in the extreme western periphery and occupied the subcontinent for a brief period of time. At the beginning of the eleventh century CE, Turkic populations bearing Islamic culture entered South Asia from Afghanistan and began spreading Islamic culture from west to east^47–57^. Outside of northern Pakistan, this series of population expansions effectively generated the gene pool from which subsequent South Asia populations developed. Yet, in northern Pakistan, appreciable movement of Islamic populations whose ultimate origins are found in the Kandahar region of southern Afghanistan did not occur until the sixteenth century^58^.

Recent genetic studies suggest that the major West Eurasian genetic contribution to South Asia derives from Neolithic Iranian and early Bronze Age steppe populations^59,60^. Other studies have further revealed contributions from Middle and Late Bronze Age steppe populations in South Asia, together with a Chalcolithic or Bronze Age Central Asian admixture scenario^61,62^. The various invasions and subsequent migrations are assumed to have resulted in major demographic expansions in the region, adding new languages and cultures to the mix of peoples already residing within the subcontinent. As a result of these processes, the majority of present-day Pakistani and Northwest Indian populations have relatively close affinities with West Eurasian populations^7,8,17,19,62–71^.

Like South Asia as a whole, the population of Pakistan encompasses a diverse array of cultures with different communities distributed into different ethnic groups. Indo-European languages are spoken by more than 70% of Pakistani ethnic groups^31,33^. These languages have been connected to the so-called “Indo-Aryan invasion” from Central Asia that occurred approximately 3.5 kya and the subsequent establishment of the caste system. The actual extent of immigration by “Aryan” populations remains controversial^31^, although at least some Indo-Iranian languages were likely introduced by immigrant Islamic groups from Afghanistan during the medieval period^72–76^. The speakers of these languages are further divided into different castes, sub-castes and tribes, reflecting the complex social organization of the region today^2,30,31,58,77–80^. Moreover, the region is home to followers of many religions, the major ones being Islam, Hinduism, Buddhism and Sikhism, with sizeable Christian and Jewish minorities also being present. All have likely contributed to the genetic and cultural diversity found in this region of South Asia.

While several studies have focused on Pakistan, genetic research on the ethnic groups in the Khyber Pakhtunkhwa Province (KPP) remains rather limited^81–87^. Thus, in this study, we conducted an extensive analysis of diversity in the mitochondrial DNA (mtDNA) and the non-recombining portion of the Y-chromosome (NRY) among members of the five major ethnic groups of Buner and Swabi Districts of KPP to elucidate their genetic history. The data generated were also compared with previously published information for geographically and ethnically diverse global populations to explore the maternal and paternal history of South Asian populations. The resulting data provide, for the first time, deep phylogeographic information about Pakistani population genetic diversity.

## Results

### Mitochondrial DNA diversity

#### Genetic lineages

The maternal genetic ancestry of 659 individuals from the five KPP ethnic groups was characterized through coding region single nucleotide polymorphism (SNP) genotyping and control region (CR) sequencing (Tables S1 and S2). A total of 54 different mtDNA haplogroups was detected among individuals of the five ethnic groups. Although sharing a number of haplogroups in common, the five populations differed significantly in the frequencies of many of these maternal lineages.

The majority (50.8%) of the identified haplogroups were of West Eurasian (WE) derivation. The most prevalent WE haplogroup was H, followed by U7, J1, W and HV. The relative proportion of these haplogroups was greatest among Gujar individuals (62.3%).

South Asian (SA) lineages were mainly represented by haplogroups deriving from macrohaplogroup M, which comprised approximately 39% of the individuals within the study populations. The most frequently occurring SA lineages were U2, followed by M3 and R5. The highest frequency of SA lineages occurred among Tanolis (47.8%).

All five ethnic groups also had a number of East Eurasian (EE) haplogroups, which together accounted for 10.2% of their mtDNAs. Haplogroup D was the most prevalent EE lineage, with A, C, F, M7, M9, M10 and Z also occurring at low frequencies. The frequency of such EE lineages was highest among Jadoons (15.2%), whereas they were far less common among Tanolis (5.2%) and Gujars (4.1%).

Certain haplogroups were confined to particular ethnic groups. For instance, J2 was present only among Gujars, while haplogroup M1 appeared only in Jadoons. Similarly, haplogroups M27, M76, R30 were only observed among Syeds, while M21, M33, M52, V and Z were nearly exclusive to Yousafzais. In addition, the more frequent haplogroups, such as D, HV, M5, M18, M30, N3, U2, U7 and W, differed considerably in their distribution among the five ethnic groups from KPP.

We further observed a high degree of CR sequence diversity among members of the five KPP ethnic groups (Table S2). Notably, between 43–74% of their mtDNAs presented unique HVS1 haplotypes. Each ethnic group also shared a modest number of mtDNA haplotypes with the other (avg = 12.8), with the Yousafzais sharing more than the other four ethnic groups. Only three mtDNA haplotypes were shared between all five ethnic groups, with these (H [#135], R5 [#196] and U7 [#284]) likely to be the founder haplotypes for the respective maternal lineages.

These patterns of diversity were mirrored in the median-joining (MJ) networks generated from HVS1 haplotypes present in the five ethnic groups (Supplemental Fig. 1). As evident from these networks, there is extraordinarily mtDNA diversity in all KPP populations. Overall, these ethnic groups shared a number of different M haplogroups, the majority being of South Asian origin, as well as a number of haplogroup U mtDNAs, including those from both South Asian and West Eurasian lineages. Haplogroup W also appeared in multiple KPP groups, haplogroup H was seen in most groups, and J, K, T and R5 comprised many of the R-derived mtDNAs in these populations.

**Figure 1:**
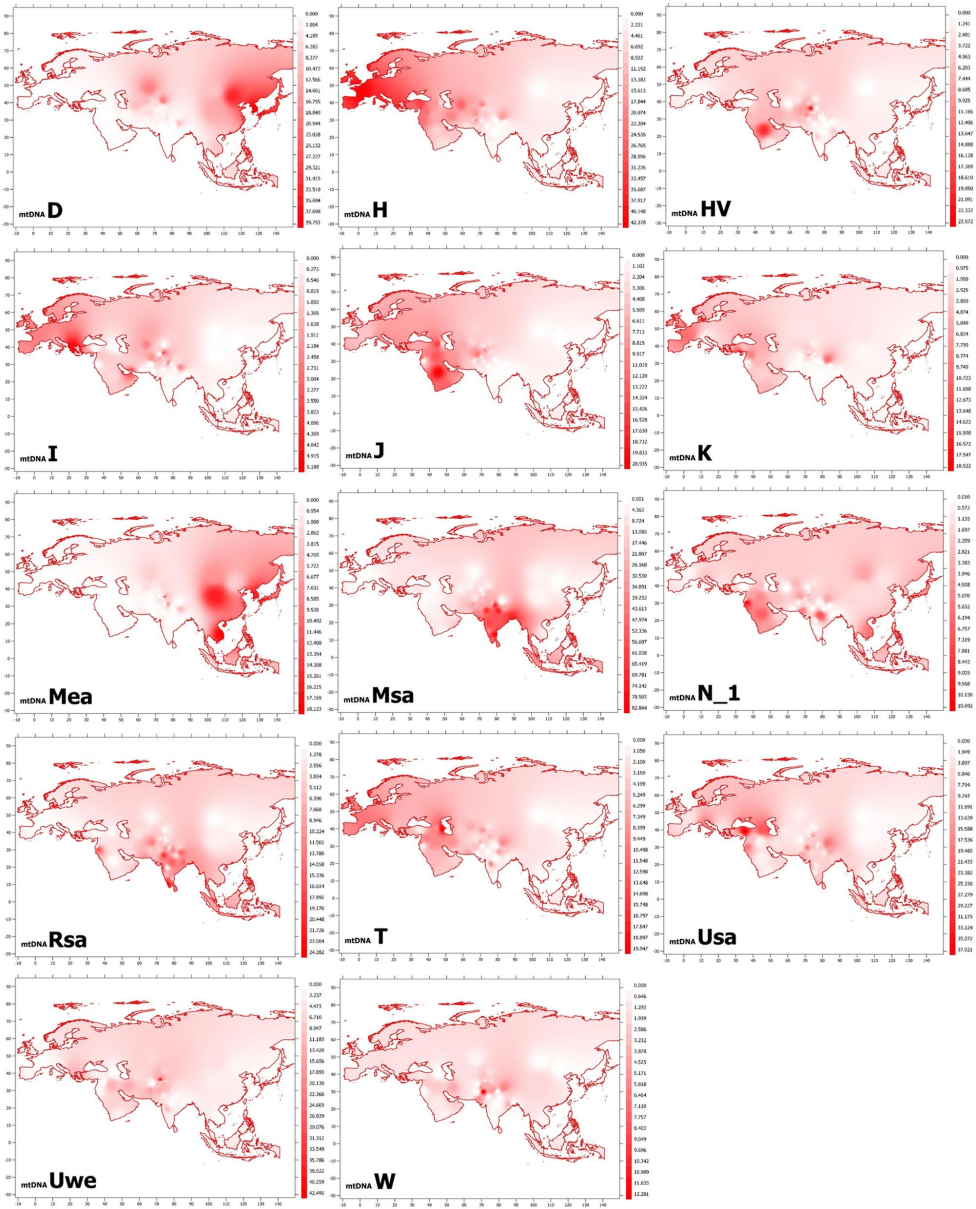
Geospatial map of mtDNA haplogroup frequencies in KPP ethnic groups and comparative populations. See the Methods section for a description of the mapping process, and Table S3 for the data on which the projection is based. With respect to the abbreviations in the different panels in the figure, “M_EA_” indicates mtDNAs belonging to East Asian haplogroups deriving from macrohaplogroup M (C, D, G, M7-M9, Z), while “M_SA_” denotes those belonging to South Asian haplogroups derived from M (M2-M10, M12, M18, etc.). Similarly, “R_SA_” indicates mtDNAs derived from R haplogroups arising in South Asia (e.g., R2, R5, R6, R7, R9, R30, R31), “U_WE_” denotes mtDNAs from U haplogroups common to West Eurasian populations (U2e, U3-U5, U7, U8), and “U_SA_” mtDNAs from U haplogroups identified in South Asian populations (U*, U1, U2a-c).

To further understand the maternal genetic background of KPP ethnic groups, we compared their mtDNA haplogroup frequency data with those from 77 Old World populations representing South Asia, Central Asia, East Asia, Middle East, Europe and the Caucasus (Table S3). We used these data and GPS coordinates associated with the populations to generate geospatial maps of mtDNA haplogroup frequencies across Eurasia. As seen in these maps (Fig. 1) and discussed above, KPP populations bear a set of maternal lineages that reflect the geographic regions from which they emerged and were dispersed over the past 40–50,000 years. Those lineages originating in East Asia (D and M_EA_) and South Asia (M_SA_) showed foci in those regions. Likewise, haplogroups having European (H, I) and Near East (HV, J, K, T, N1) origins were concentrated in those areas, although clearly having been spread into South Asia since evolving. The other lineages (U_SA_, U_WE_, W) were somewhat less concentrated in any one region. While providing insights into the distribution of these haplogroups across Eurasia, this analysis may have been affected by the sample sizes, hence, the relative haplogroup frequencies, used for the comparative populations.

#### Genetic variation and relatedness of KPP populations with other Asian groups

Summary statistics describing genetic diversity in the five KPP ethnic groups and other Pakistani populations are shown in Table 1. All five KPP groups exhibited great haplotypic diversity, with the highest occurring in Yousafzais (0.994) and the lowest among Tanolis (0.970). Nucleotide diversity estimates were also essentially the same for KPP and other Pakistani populations. Neutrality tests yielded significantly negative Tajima’s D and Fu’s F estimates for the KPP populations, suggesting that they experienced relatively recent expansions in population size.

**Table 1:** Summary statistics for KPP ethnic groups and other Pakistani populations based on mtDNA HVS-1 haplotypes.

Population	n	#	HD	ND	PD	RI	Tajima’s D	Fu’s FS
Gujars	122	56	0.975	0.022 ± 0.012	5.71 ± 2.76	0.016	−1.805	−25.194
Jadoons	99	66	0.988	0.022 ± 0.012	5.67 ± 2.74	0.011	−2.003	−25.268
Syeds	127	93	0.992	0.023 ± 0.012	5.89 ± 2.83	0.009	−2.094	−25.117
Tanolis	134	59	0.970	0.022 ± 0.012	5.76 ± 2.78	0.012	−1.664	−25.147
Yousafzais	177	131	0.994	0.022 ± 0.012	5.61 ± 2.71	0.011	−2.107	−25.055
Kashmiri	317	211	0.993	0.023 ± 0.012	5.89 ± 2.82	0.011	−2.117	−24.675
Makrani	100	60	0.984	0.027 ± 0.014	7.01 ± 3.32	0.006	−1.742	−24.936
Pathan	230	153	0.992	0.022 ± 0.012	5.61 ± 2.70	0.010	−2.225	−24.934
Saraiki	85	47	0.957	0.023 ± 0.012	5.98 ± 2.88	0.013	−1.637	−25.238
Sindhi	115	81	0.992	0.024 ± 0.013	6.16 ± 2.95	0.013	−1.579	−25.069
Balti	49	32	0.979	0.020 ± 0.019	5.12 ± 2.53	0.016	−1.772	−22.570
Bangash	25	17	0.973	0.012 ± 0.011	5.10 ± 2.56	0.045	−1.162	−7.036
Khattak	25	14	0.932	0.021 ± 0.011	5.34 ± 2.66	0.073	−0.413	−2.867
Mahsuds	25	10	0.917	0.019 ± 0.011	4.96 ± 2.50	0.028	−0.680	−0.142
Orakzai	25	18	0.967	0.022 ± 0.012	5.61 ± 2.79	0.045	−1.186	−7.811
Brahui	38	22	0.952	0.018 ± 0.010	4.63 ± 2.32	0.032	−1.619	−10.643
Hazara	23	21	0.992	0.022 ± 0.012	5.76 ± 2.86	0.018	−1.638	−15.567
Hunza	44	32	0.980	0.024 ± 0.013	6.13 ± 2.97	0.016	−1.912	−21.877
Kalash	44	11	0.830	0.015 ± 0.009	3.86 ± 1.98	0.064	−0.041	−0.249
Parsi	44	20	0.943	0.018 ± 0.010	4.53 ± 2.27	0.058	−1.501	−6.759

n = number of samples; # = number of haplotypes; HD: Haplotype Diversity; ND: Nucleotide Diversity; PD: Pairwise differences; RI: Raggedness index; Citations and references for the comparative populations are provided in Table S9.

**Figure 2:**
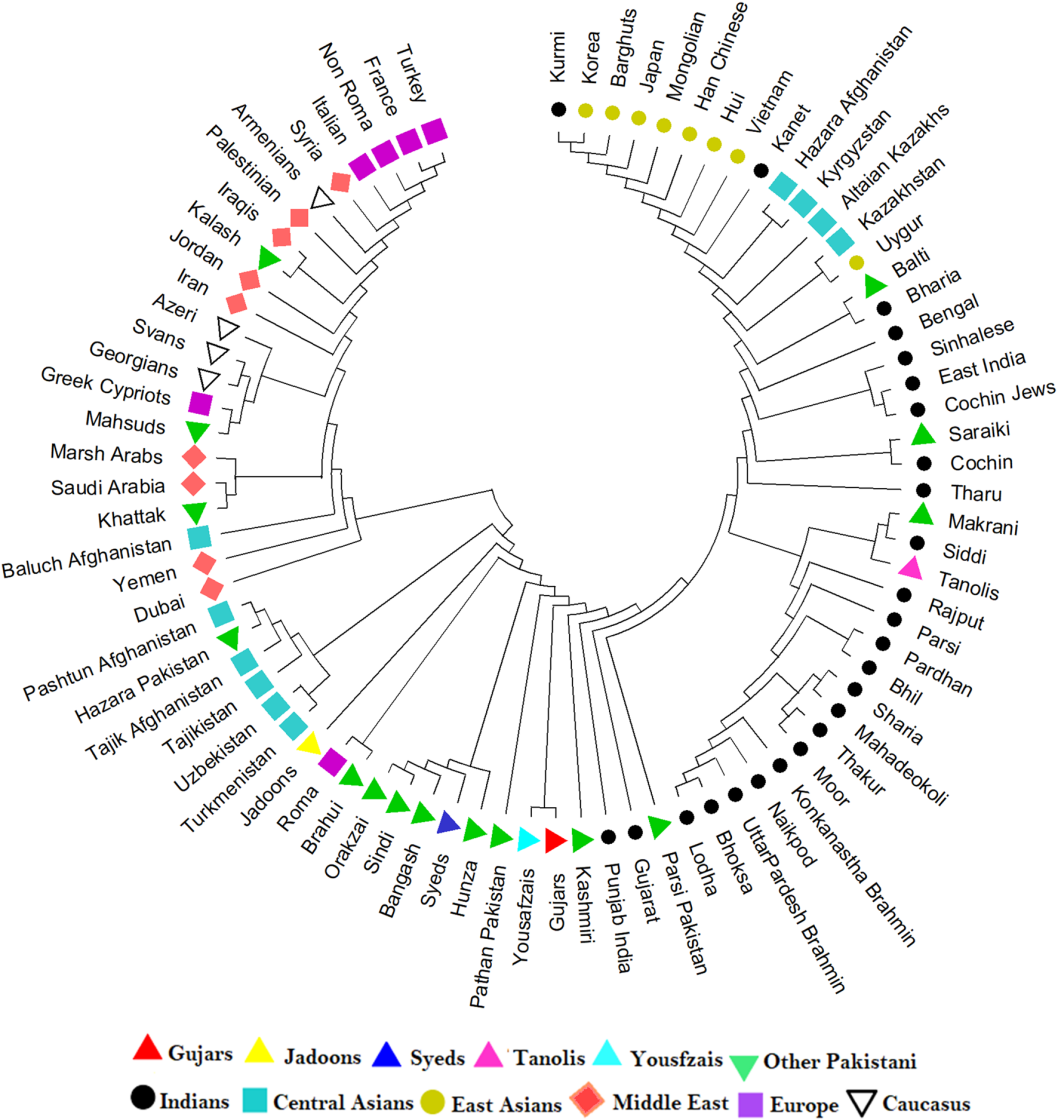
A Neighbor-Joining tree showing the genetic relationships between KPP ethnic groups and 77 world populations based on F_ST_ estimates from mtDNA HVS1 sequence data (Table S4).

To further elucidate the maternal genetic relationships between the five KPP ethnic groups and comparative populations, we generated pairwise F_ST_ values based on their HVS1 sequences (Table S4). The resulting estimates were then visualized in a Neighbor-joining (NJ) tree (Fig. 2). In the NJ tree, Jadoons, Syeds, Yousafzais and Gujars clustered together with the majority of Central Asian and KPP populations, but were clearly distinguished from Middle Eastern, Indian, East Asian, European and Caucasus populations. By contrast, the Tanolis were more closely positioned with Indian populations.

Analysis of molecular variation (AMOVA) was conducting using F_ST_ estimates based on HVS-1 haplotypes in KPP and comparative populations. In this analysis, 95.2% of the genetic variation occurred within all 82 Pakistani and comparative populations (Table 2). When grouping populations by country of origin or region, the genetic variation among countries or region accounted for only 2.6% of the variation, whereas 2.55% was explained by the differences between population samples within countries. A similar trend was observed for the AMOVA results based on ethnicity. Thus, KPP ethnic groups were not strongly differentiated from each other or regional populations in terms of their maternal lineage composition.

**Table 2:** AMOVA results for mtDNA HVS-1 sequences in KPP and comparative populations.

No Groups						
Source of variation	d.f.	Sum of squares	Variance component	% of variance	Fixation Indices	P-value
Among populations	72	1782.704	0.15213 Va	4.79	F_ST_: 0.04786	0.000 ± 0.000
Within populations	10,496	31,766.373	3.02652 Vb	95.21		
Total	10,568	33,549.077	3.17865			
**Grouped by Geography**						
Among groups	6	865.080	0.08299 Va	2.60	F_SC_ (Va):0.026	0.000 ± 0.000
Among populations within groups	66	917.624	0.08129 Vb	2.55	F_ST_ (Vb):0.051	0.000 ± 0.000
Within populations	10,496	31,766.373	3.02652 Vc	94.85	F_CT_ (Vc):0.026	0.000 ± 0.000
Total	10,568	33,549.077	3.19080			
**Grouped by Ethnicity**						
Among groups	15	977.099	0.08197 Va	2.57	F_SC_ (Va): 0.025	0.000 ± 0.000
Among populations within groups	57	805.604	0.07913 Vb	2.48	F_ST_ (Vb): 0.051	0.000 ± 0.000
Within populations	10,496	31,766.373	3.02652 Vc	94.95	F_CT_ (Vc): 0.026	0.000 ± 0.000
Total	10,568	33,549.077	3.18762			

### Y-Chromosome diversity

#### Genetic lineages

The analysis of NRY variation in 678 male individuals from the same ethnic groups yielded 11 distinct haplogroups defined by 54 SNP and 19 Y-STR markers (Tables S5 and S6). The majority of their Y-chromosomes fell into one of four haplogroups, namely, R1a1a-M17 (50%), R1b1a-M297 (17.4%), O3-M122 (13.9%) and L-M20 (7.1%) with another seven haplogroups comprising the remaining 11.6%. The haplogroup profiles of the five ethnic groups suggested that the genetic diversity in these groups was structured along ethnic boundaries.

*West Eurasian Lineages.* The most common paternal haplogroup in the study populations was R1a1a-M17. It appeared at high frequencies among individuals of three of the five ethnic groups (Syeds, Yousafzais and Gujars). R1a1a-M17 is also one of the most common haplogroups in Eurasia, with high frequencies occurring in Eastern Europe, Central Asia, and South Asia^8,43,88–94^. In neighboring Afghanistan, R1a1a-M17 is frequent among Pashtuns (51.02%) and Tajiks (30.36%) but less so in Uzbeks (17.65%) and Hazaras (6.7%)^95^. In addition, it has been observed at high frequency (~ 80%) among Yousafzais of Swat Pakistan^82^, a finding consistent with our data.

The second most common haplogroup was R1b1a-M297. It occurred in the Tanolis at a very high frequency but appeared at very low frequencies in the Jadoons, Yousafzais and Syeds, while being completely absent in the Gujars. Haplogroup R1b is the most frequent Y-chromosome lineage in Western Europe (> 70%)^96–99^, but also appears in South Asian populations at modest frequencies^44,100^.

The other West Eurasian haplogroups appeared non-uniformly in the study populations. G2a-P15 and I2-P215 occurred at low frequency in only the Yousafzais. G2a is thought to have arisen in Anatolia and the Caucasus^101^, and may be associated with the Neolithic expansion throughout the region^94^. G2a has also been observed throughout the Near East^102^ and Mediterranean region^103^, and occurs in South Asia in appreciable frequencies^44,95,104^. By contrast, I2-P215 may have arisen in the Balkans and central Europe^105^, since it is commonly observed in Slavic speaking populations of southern Europe^106^.

Gujars, Jadoons and Yousafzais exhibited haplogroup J2a-M410 at low frequencies, while J2b-M12 occurred at low frequency in the Gujars and Yousafzais. Previous studies have demonstrated that J2a-M410 and J2b-M12 are associated with the demic diffusion of Neolithic farmers in North Africa and Eurasia from Mesopotamia (Iraq and Syria)^107–109^. Both J2a-M410 and J2b-M12 (0–8%) also appear at low frequencies in populations inhabiting different parts of India^110^. In general, the presence of J2a-M410 and J2b-M12 in Pakistan and India has been considered indicative of gene flow from western Asia^43,44^.

*South Asian Lineages.* Haplogroup H-M69, which is commonly observed in South Asia^60,111,112^, occurred in all five ethnic groups at modest frequencies. The Gujars also had a moderate frequency of haplogroup L-M20, with this paternal lineage being present at low frequency among Syeds, Yousafzais, and Jadoons and completely absent in Tanolis. Haplogroup L commonly occurs in populations from Pakistan, India and Afghanistan, and has spread into the Near East and Iran^44,84,94,113^. In addition, South Asian-specific haplogroup R2-M124 occurred at low frequencies among all KPP populations. Haplogroup R2-M124 has mainly been found in Indian, Iranian, Pakistani and Central Asian populations, and postulated to have a Central Asian origin^8,44,94,114–118^.

*East Eurasian Lineages.* East Eurasian haplogroup O3-M122 occurred nearly exclusively in Jadoons, and otherwise appeared at very low frequency in Yousafzais, Gujars and Tanolis while being absent in Syeds. O3-M122 is the most common haplogroup among Han Chinese populations, occurring at frequencies of 50–60% in them^119–121^. It also occurs at very low frequencies in India and Pakistan, mostly likely due to the westward expansion of Tibeto-Burman speakers into South Asia^44^.

By contrast, all five KPP populations possessed Q-MEH2 Y-chromosomes at low frequencies. The MEH2 marker occurs downstream of the M242 marker that helps to define this paternal lineage. Haplogroup Q-M242 probably originated in Central Asia and has been distributed widely in Northeast Asia, while also appearing at low frequencies in Europe and the Middle East, mostly likely to due to the influence of Mongolic and Turkic speaking populations^93,122^. Among the Pashtuns of Afghanistan, the frequency of haplogroup Q-M242 is about 18.4%^95^.

These patterns of diversity were mirrored in the median-joining (MJ) networks generated from Y-STR haplotypes present in the five ethnic groups (Supplemental Fig. 2). Gujars, Syeds and Yousafzais all exhibited specific R1a lineages, although sharing some with other KPP ethnic groups in which other haplotypes from this paternal lineage also occurred. In addition, the Tanolis showed a wide range of R1b haplotypes, indicating their centrality to the paternal gene pool for this population. Similarly, Jadoons had mainly O3 haplotypes that appeared in a starlike cluster suggestive of an older founder event, whereas L haplotypes were dispersed among all ethnic groups with no specific pattern of clustering.

**Figure 3:**
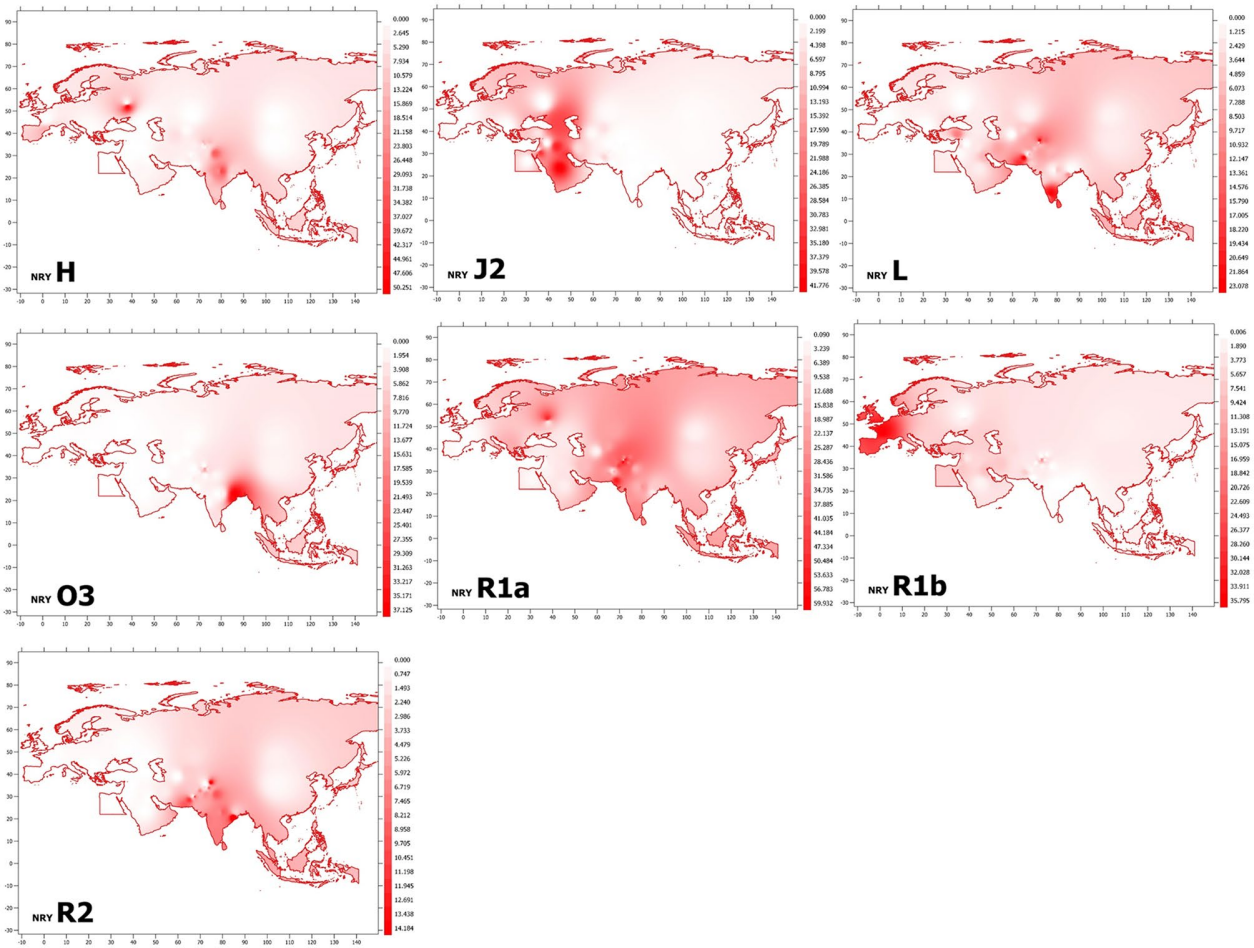
A geospatial map of NRY haplogroup frequencies in KPP ethnic groups and comparative populations. See the Methods section for a description of the mapping process, and Table S6 for the data on which the projection is based.

To further assess the paternal genetic background of KPP ethnic groups, we compared their NRY haplogroup frequencies with those from 82 Old World populations representing South Asia, Central Asia, East Asia, Middle East, Europe and the Caucasus (Table S7). We used these data and GPS coordinates associated with the respective populations to generate geospatial maps of NRY haplogroup frequencies across Eurasia. As seen in these maps (Fig. 3) and discussed above, KPP populations bear a set of paternal lineages that reflect the geographic regions from which they emerged and were dispersed into adjacent area at different points in time. Haplogroups H, L and R2 clearly have South Asian roots, with J2 arising in the Near East and O3 in South-East Asia, as previously above. Similarly, R1b appears to have arisen and spread into South Asia from Europe, while R1a shows a more complex pattern reflective of its dual origin in Eurasia. Thus, NRY diversity in KPP populations reveals these prehistoric expansions of paternal lineages into South-Central Asia, while also reflecting more recent population movements and ethnic group formation, as described below.

#### Genetic variation and relatedness of KPP populations with other Asian groups

Molecular diversity estimates were calculated from Y-STR haplotypes in an effort to quantify the degree of paternal genetic variation in the five KPP ethnic groups and other Pakistani populations (Table 3). Gene diversity estimates showed that Gujars were more diverse than the other four ethnic groups, with Syeds being the least diverse by far. Unlike the mtDNA data, NRY pairwise differences were greater among the Gujars than individuals of the other four ethnic groups.

**Table 3:** Summary statistics for KPP ethnic groups and other Pakistani populations based on Y-STR haplotypes.

Populations	n	#	HD	PD
Gujars	124	37	0.910	5.47 ± 2.65
Jadoons	114	36	0.796	3.07 ± 1.61
Syeds	129	31	0.794	3.05 ± 1.60
Tanolis	134	32	0.795	3.21 ± 1.67
Yousafzais	177	85	0.905	4.32 ± 2.15
Yousafais_Old	146	90	0.966	5.07 ± 2.48
Gujars_Swat	20	10	0.758	5.29 ± 2.67
Kohestani	20	14	0.890	6.13 ± 3.04
Tarklani	20	9	0.837	2.65 ± 1.47
Utmankhail	20	6	0.684	2.27 ± 1.30
Yousafzais_Swat	20	10	0.800	3.43 ± 1.83
Pathan_Pakistan	270	152	0.973	5.61 ± 2.70
Kashmiri	101	68	0.981	6.38 ± 3.05
Hazara_Pakistan	153	73	0.910	4.15 ± 2.07
Punjabi	394	266	0.995	6.10 ± 2.91
Sheikh	180	100	0.984	6.15 ± 2.94
Gujars_Punjab	176	84	0.971	6.23 ± 2.97
Baluch	59	48	0.988	6.68 ± 3.20
Brahui	110	80	0.968	5.66 ± 2.73
Burusho	86	55	0.990	6.17 ± 2.96
Kalash	44	23	0.946	5.55 ± 2.72
Makrani	58	52	0.996	6.75 ± 3.23
Parsi_Pak	90	56	0.969	6.09 ± 2.93
Sindhi	122	97	0.990	5.97 ± 2.87

n = number of samples; # = number of haplotypes; HD: Haplotype Diversity; PD: Pairwise differences; Citations and references for the comparative populations are provided in Table S10.

**Figure 4:**
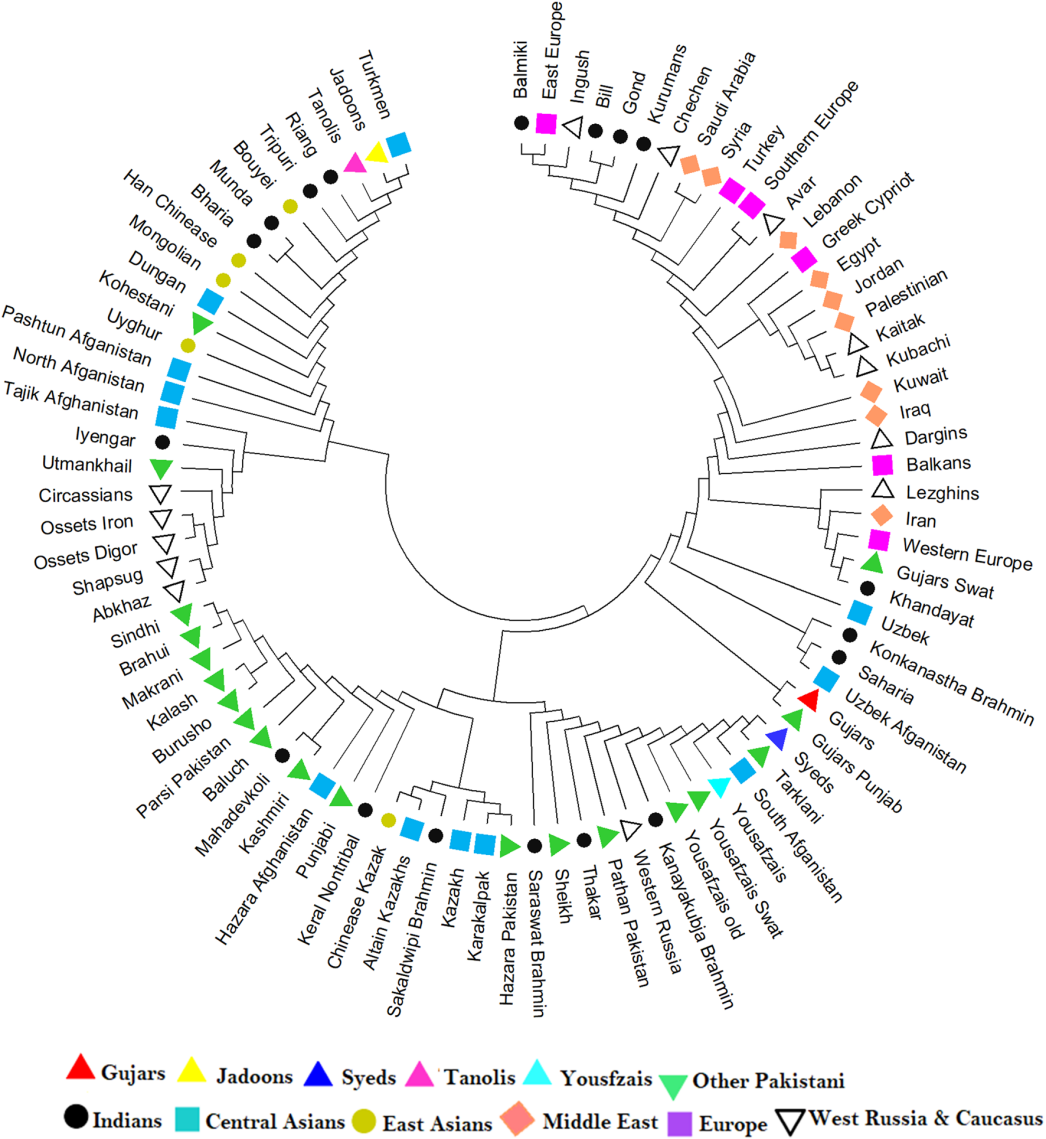
A Neighbor-Joining tree showing the genetic relationships between KPP ethnic groups and 82 world populations based on R_ST_ estimates from Y-STR haplotype data (Table S8).

We compared the Y-STR data from the five KPP ethnic groups with those from the comparative populations to place their genetic diversity in a broader context. Y-STR haplotypes for this analysis were reduced to 10-loci haplotypes in order to incorporate data from as many comparative populations as possible. Subsequently, we calculated pairwise R_ST_ values (Table S8) and visualized them in a NJ tree (Fig. 4).

The NJ tree revealed different genetic affinities of the KPP populations. Gujars from our study clustered tightly with a Gujars population from Punjab^123^, Yousafzais from this study clustered closely with populations from South Afghanistan^104^ and other Yousafzai groups^82,83^. In addition, the Syeds from our study were positioned close to a Tarklanis population from the Dir District of Pakistan^82^. By contrast, Jadoons and Tanolis generally clustered somewhat near each other and with Turkmen population from Central Asia and ethnic groups from northeast India.

We conducted an AMOVA using R_ST_ estimates generated from Y-STR haplotypes in KPP and comparative populations (Table 4). This analysis indicated that the great majority of Y-chromosome diversity occurs within populations (81.8%), with less than 20% occurring between groups across the 87 populations considered. When categorized by geography, genetic variance accounted for 3.8%, whereas 14.8% of total variance was explained by differences between population samples within geographic regions. These estimates were approximately the same for the AMOVA based on ethnicity. Thus, KPP and comparative populations were moderately genetically differentiated from each other based on their paternal lineages.

**Table 4:** AMOVA results for Y-STR haplotypes in KPP and comparative populations.

No Groups						
Source of variation	d.f.	Sum of squares	Variance component	% of variance	Fixation Indices	P-value
Among populations	79	18,852.353	1.59547 Va	18.23	F_ST_: 0.182	0.000 ± 0.000
Within populations	11,641	83,324.558	7.15785 Vb	81.77		
Total	11,720	102,176.911	8.75333			
**Grouped by Geography (Ten Groups: Country or Region)**						
Among groups	9	6701.154	0.33529 Va	3.81	F_SC_ (Va):0.153	0.000 ± 0.000
Among populations within groups	70	12,151.198	1.29605 Vb	14.75	F_ST_ (Vb):0.186	0.000 ± 0.000
Within populations	11,641	83,324.558	7.15785 Vc	81.44	F_CT_ (Vc):0.038	0.000 ± 0.000
Total	11,720	102,176.911	8.78919			
**Grouped by Ethnicity**						
Among groups	14	8879.462	0.44807 Va	5.10	FSC (Va):0.141	0.000 ± 0.000
Among populations within groups	65	9972.890	1.17945 Vb	13.43	FST (Vb):0.185	0.000 ± 0.000
Within populations	11,641	83,324.558	7.15785 Vc	81.47	FCT (Vc):0.051	0.000 ± 0.000
Total	11,720	102,176.911	8.78537			

## Discussion

Previous genetic research on Pakistani populations has largely been limited to studies of either Y-STR or mtDNA variation in a single ethnic group^83,85–87,123–131^, or Y-chromosome and mtDNA analysis in many ethnic groups with limited sample sizes^81,82,84,132^. None of these studies broadly analyzed genetic diversity among the myriad ethnic groups residing in Pakistan. This study provides the first survey of mtDNA and NRY variation in members of five major ethnic groups inhabiting Buner and Swabi Districts of KPP.

The paternal and maternal gene pools of the KPP populations were found to consist of West Eurasian, South Asian and East Asian lineages. However, the patterns of mtDNA and NRY diversity amongst these populations are fairly different, suggesting contrasting paternal and maternal genetic histories for them. Based on mtDNA data, Syeds, Yousafzais and Jadoons have close affinities to one another, while NRY data reveal close affinities between Gujars, Syeds and Yousafzais. Overall, Yousafzais show greater genetic affinities with the Syeds than to any of the other three ethnic groups, whereas Tanolis, Jadoons and Gujars were outliers in the NJ trees relative to the other KPP populations, depending on the data set being analyzed. Such results are not entirely surprising, given that Yousafzais and Syeds claim to be Afghans, or at least in the latter case “Arabs.” By contrast, Gujars are not considered Pathans at all, nor are Tanolis, while Jadoons are allegedly descendants of Pashtun leader Ghurghusht, the third and youngest son of Qais^58^. We elaborate on the histories of these ethnic groups below.

Yousafzais share more mtDNA haplotypes with all other ethnic groups than any of the other four and share the most with Syeds. This observation likely reflects both the larger sample size and the effective population size for Yousafzais. These data also suggest some degree of gene flow between these populations, or else the selection of marriage partners whose mtDNAs draws from the common maternal gene pool for South-Central Asia that developed over time^7,33,63,133,134^. The resulting diversity is now being resorted and reshuffled within extant ethnic populations.

The MJ networks of the major common NRY haplogroups show that flow of paternal lineages among the five ethnic groups is quite limited and consistent with high levels of male endogamy practiced by them. Similar Y-chromosome results have been previously reported for Central Asian and South Asian ethnic groups^44,82,91,100,135,136^, but with less pronounced genetic differentiation in maternal lineages^135–138^.

These findings are consistent with evidence from historical and ethnographic research involving populations from this region. According to Barth^80^, there has been relatively little intermarriage between any of the members of these ethnic groups. They tend to live in isolation relative to other ethnic groups and discourage intermarriage between them. However, as Barth notes^80^, “the Pathans allow marriages of equals, even when close relatives, and the giving of a daughter to a man of superior status but discourage the giving of a woman in marriage to inferior men. Landowners, as a group, thus, tend to marry endogamously but they also take some women in marriage from lower groups, whereas they will not give up their daughters in marriage to inferiors.” These practices may possibly have shaped the population dynamics of KPP populations and led researchers to describe local populations as being genetically isolated and marked by high levels of inbreeding^139–141^.

Such an explanation overlooks two important facts, though. First, as devout Muslims, Pathans believe that all individuals are equal in the eyes of the creator. Consequently, there is no absolute genealogical “litmus test” of worthiness equivalent to the Hindu notion of inborn purity and pollution^142,143^. Second, in the political sphere, Pathans are extremely competitive, and Pathan chiefs tend to spend far beyond the revenue generated by their landholdings^144^. Because of these two factors, economic advantage can outweigh inherited social status in arranging marital partners, especially when village leaders are seeking to consolidate their power in the political arena^80,142,145–149^.

With respective to the different KPP ethnic groups, Gujars are characterized by having predominantly R1a1a-M17 Y-chromosomes, the frequency of which is the highest observed among the populations of the Indus Valley^89^. Otherwise, they are marked by 30% SA haplotypes and a low frequency of EA haplotypes. They also have a high frequency of South Asian haplogroup L-M20 compared to other KPP populations, supporting their historically documented affinities with various South Asian ethnic groups^150–153^, especially those residing in the northwestern portion of South Asia^154^.

Gujar maternal ancestry is largely congruent with their paternal genetic ancestry. Their mtDNAs are largely of WE origin, although some derive from SA and EA^81^. Gujars also possess haplogroups linking them with West Asia (HV, U7, W) while having relatively few EA mtDNAs. Based on this pattern of diversity, they show strong genetic affinities with the Yousafzais, Kashmiri and other Pakistani populations.

With respect to their origins, one hypothesis proposes that Gujars expanded into India from Central Asia, while another suggests they came from Georgia via Afghanistan in the fifth century CE. Based on our data, Gujars generally resemble Iranians who mixed with local populations rather than populations from the Caucasus^155^. By contrast, Gujars of Jammu and Kashmir show mtDNA affinities with populations from Uttar Pradesh and Arunachal Pradesh to the east^156^. Thus, our data suggest a more complex origin for KPP Gujars. One scenario could involve an indigenous population with genetic affinities to geographically proximate Jats and Rajputs mixing with Indo-Iranian or Turkic speaking Muslim populations, and then migrating into the region from the steppes of Central Asia^157^.

Unlike other KPP ethnic groups, the Jadoons exhibit a strongly East Asian paternal ancestry, with NRY haplogroups O3-M122 and Q-MEH2 representing 82.5% of their Y-chromosomes. Although O3-M122 is very rare among South Asian populations^44^, Q-M242 appears at modest frequencies in them. In the NRY NJ tree, the Jadoons occupy an outlier position relative to other KPP populations, but exhibit affinities with Turkmen from Central Asia. In this regard, Mongol expansions into Central-South Asia probably brought NRY haplogroups C3, O3, and Q to Pakistan during the medieval period, and NRY diversity in Kazakh populations from Central Asia was probably similarly influenced during this time^94,158,159^. The rest of their Y-chromosomes belong to either WE or SA haplogroups, and appear similar to types present in other KPP populations, suggesting some degree of gene flow between them.

Jadoons mtDNA shows greatest similarity to groups from WE followed by SA with less affinity to EA groups. As such, Jadoons exhibit a pattern of extra-regional affinities that are generally like those observed among Gujars, Syeds and Yousafzais. The neighbor-joining tree also identifies affinities to European Roma populations and to other South-Central Asian groups. As such, these results corroborate previous studies that identify a genetic affinity of Roma populations to South Asian groups, especially those residing in the northwestern region of the subcontinent^160,162^. Viewed as a whole, genetic diversity among Jadoons appears to reflect a scenario in which male-mediated gene flow into the region was followed by these immigrant males subsequently marrying indigenous females thereby yielding a maternal gene pool similar to those possessed by members of other Pakistani and Central Asian ethnic groups.

Most Syeds possess NRY haplogroup R1a1a-M17, along with a unique array of Y-STR haplotypes of this patrilineage that is coupled with low prevalence of Y-chromosomal variations common to South and East Asians. This combination aligns Syeds with other Pakistani and Central Asian ethnic groups while distancing them from ethnic groups of the rest of the subcontinent and East Asia. Matrilineal genetics yield a similar pattern. Syeds are marked by high frequencies of WE lineages coupled with low frequencies of lineages common to South and East Asians. While this pattern aligns Syeds with other Pakistani and some Indian Samples^7,63,85,87,118,125–127,132^, they are distinct through their hgh frequencies of haplogroup U2, T2, M9, X and R30 mtDNAs.

Although Syeds are hypothesized to have come from the Near East and entered South and Central Asia during the Mongol invasions of the thirteenth and fourteenth centuries, mtDNA and NRY data instead support a scenario in which Syeds have an ultimate origin in Afghanistan coupled with long-standing gene flow with Central Asian populations^163^. Moreover, the topography of northern Pakistan with its formidable mountains and narrow, steep-sided valleys may have fostered the establishment of localized endogamous social groups that, over time, developed into largely reproductively isolated distinct ethnic groups.

This explanation corroborates results obtained in other mtDNA studies from South Asia^63,85,132^. In which populations residing west of the Indus Valley possess mtDNA lineages largely of West Eurasian derivation with limited contributions from South Asia and East Eurasia, while those found to the south and to the east are characterized by mtDNA profiles that feature higher frequencies of deep-rooted lineages indigenous to South Asia^63^. Likewise ethnic groups from KPP are marked by generally show close affinities to one another, but share only distant affinities to populations from Iran, Uzbekistan and Kazakhstan^81,85^. Sharma et al.^134^ further noted the mtDNA divergence between ethnic groups of Jammu and Kashmir in northern India to be greater than within Pakistani groups or populations from Europe and the Caucasus. Such results not only document the limited impact of the medieval incursion of different Pashtun ethnic groups from Afghanistan and the Iranian Plateau into the northwestern periphery of South Asia, but also suggest that such introgressive events involved non-local individuals of both sexes, rather than being limited to males^134,164^.

Tanolis, whose communities are restricted to hilly area of Swabi District along the border with Buner and Haripur Districts of KPP, have predominantly R1b1a-P297 Y-chromosomes, along with a low frequency of SA and EA haplotypes. Based on several studies, haplogroup R1b is thought to have spread with pastoralism and Indo-European speakers into South Asia^165–168^. For this reason, the Tanolis are relatively dissimilar to other KPP and comparative populations. From a mtDNA perspective, Tanolis have a high frequency of haplogroup N3, which arose in Western Eurasia, as well as higher frequencies of SA haplogroups such as M2-M6 than other populations. As a result, Tanolis show genetic similarities with Siddi^169^ and other populations from India.

Given this genetic profile, Tanolis may be an outlier within the Indo-Pakistani subcontinent. While suggested to have Turkic roots, and also claiming Pashtun ancestry tracing to the fifteenth century CE, the Tanolis appear to have a different genetic origin than the other four KPP ethnic groups. It is possible that they have experienced significant genetic drift, perhaps due to founder effects, which would affect the frequencies of their paternal lineages. Yet, the latter scenario is not consistent with the high proportion (47.8%) of South Asian mtDNA lineages observed in Tanolis relative to other KPP ethnic groups.

Yousafzais are the most genetic diverse KPP population in this study. While exhibiting a high frequency of R1a1a Y-chromosomes, they also have a mixture of other NRY haplogroups, including West Eurasian G2, I2, J1 J2, South Asian H, L, R2 and East Asian O3, Q. Four of these haplogroups (G2a, R1a, J2a1b, I2a) are likely associated with male-mediated migrations related to Neolithic farming^45,98,101,170,171^. They also exhibit genetic affinities with other Yousafzais populations^82,83^. The Yousafzais also exhibit considerable mtDNA diversity. Their maternal lineages are largely of WE derivation, with moderate frequencies of SA and low frequency of EA haplotypes also being present. Based on these data, the Yousafzais show genetic similarities to Central Asian and other Pakistani populations.

Overall, Yousafzais are marked by affinities to local non-Pathan groups both paternally and maternally. Such findings suggest that Yousafzais absorbed a number of local males, perhaps through religious conversion of the most successful landholders^80^, and also integrated local females into their population, either as spouses and daughters of local non-Pathan converts or through hypergamous unions with Yousafzai men^80^. Both avenues of gene flow are well-documented throughout South Asia^55,145,146,172–175^, including regions of northern Pakistan such as Gilgit-Baltistan^147^, most likely reflect endogamous practices that involved the assimilation of foreign females into the populations.

## Conclusions

As described above, the patterns of mtDNA and NRY diversity amongst the KPP ethnic groups are fairly different, suggesting contrasting paternal and maternal genetic histories for them. We have attempted to situate these data in the context of archaeological, ethnographic, historical, genetic, and linguistic evidence to better explain the complex pattern of ethnic diversity in Pakistan and the KPP region. Yet, as shown in this genetic analysis, there are many uncertainties in the population histories of these ethnic groups. Future analysis of mitogenome and whole genome sequences will greatly facilitate the testing of the hypothesized origins and biological relationships of KPP populations outlined in this study.

## Materials and methods

### Sample and data collection

**Figure 5:**
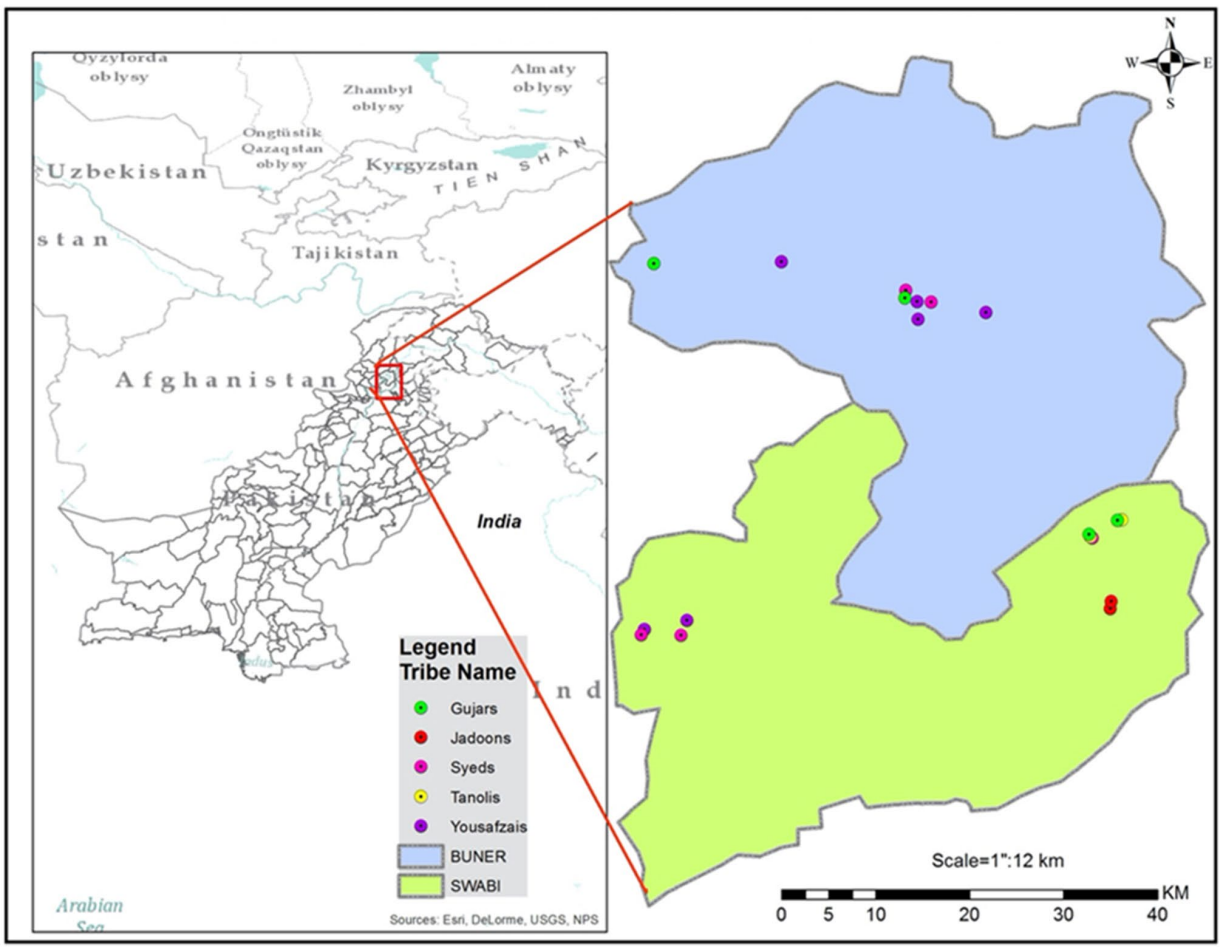
A map of Pakistan showing the locations of fieldwork in the Khyber Pakhtunkhwa Province. DNA samples were collected from areas in which each ethnic group was highly concentrated. Gujars, Syeds and Yousafzais were sampled from both Buner and Swabi Districts, while the Jadoons and Tanolis were only sampled in the Swabi District. The map was created with the ArcGIS software, v10.3.1., based on source map from ESRI https://www.esri.com/en-us/home.

Ethnographic fieldwork and sample collection were undertaken in 13 villages located within Buner and Swabi Districts of KPP in 2014–15 (Fig. 5). Within the Buner District, the villages included Bajkata, Channar Swari, Dewana Baba, Kingargalai, Sonigram, Swari Bazar, Takhtaband, while in Swabi District they included Dalori Gadoon, Dobyan, Gani Chatra, Kabgany Gadoon, Utla and Yar Hussain. A total of 700 unrelated male volunteers from five endogamous ethnic groups were the subjects of this research effort. Prior to starting this study, the Institutional Biomedical Ethics Committee of Hazara University Mansehra reviewed the project details and approved the protocol for obtaining informed written consent from study participants (ref # 73/HU/ORIC/IBC/2013). All experimental procedures were carried out in accordance with the approved guidelines of the Research Ethics Committee of Hazara University Mansehra. This research was also approved by the Institutional Biomedical Ethics Committee, Islamia College Peshawar (ref #530/ORIC/ICP) and the University of Pennsylvania IRB #8.

### DNA analysis

#### Genomic DNA preparation

Saliva samples were collected from all participants with informed consent written in English and Urdu. Genomic DNAs were isolated from the buccal cell samples using a modified protocol of Aidar and Line^176^. DNA concentrations were measured with a NanoDrop ND-1000 spectrophotometer and normalized to 1 ng/µl.

#### Mitochondrial DNA analysis

Maternal genetic ancestry was elucidated through analysis of mtDNA variation. A total of 659 individuals from five ethnic groups were surveyed for mtDNA variation. All samples were screened for phylogenetically informative single nucleotide polymorphisms (SNPs) defining the major branches (haplogroups) of the human mtDNA phylogeny with custom TaqMan assays^177–179^. All TaqMan assays were read on an ABI Prism 7900 HT Fast Real-Time PCR System. SDS v2.3 was used to analyze all runs, and the resulting allelic calls checked through visual inspection.

Samples were then surveyed for sequence variation through control region (CR) sequencing using published methods^177–179^. All variable positions were determined relative to the revised Cambridge Reference Sequence (rCRS)^180,181^. The CR sequence data defined maternal haplotypes in these individuals, and all haplogroups were ascertained relative to existing mtDNA databases, such as Phylotree version 17^182,183^. A single PCR–RFLP test was also used to screen mtDNA samples for the 14,766 SNP that characterizes haplogroup HV.

#### Y-chromosome analysis

Paternal genetic ancestry was elucidated through analysis of NRY variation in male participants. The DNAs of 678 individuals were screened for phylogenetically informative SNPs in a hierarchical fashion according to published information and previously published methods^93,184–186^. The SNP genotyping involved 47 custom TaqMan assays that were read using the ABI 7900HT Fast Real-Time PCR System^177–179^. They were then additionally surveyed for variation at 17 Y-STR loci using the ABI Y-filer Kit, as previously described^177–179^. Two other STR loci, along with six insertion–deletion (indel) SNPs, were genotyped in a separate custom multiplex assay^177–179^. The multiplex PCRs were read on an ABI 3130xl Genetic Analyzer with POP-4 polymer using GeneScan™-500 LIZ™ size standards. The resulting data were analyzed using GeneMapper1 ID Software v3.2. STR allele sizes were identified based on previous recommendations^187^. Quality control procedures included checking SNP genotypes for phylogenetic consistency and comparing the data with haplogroups predicted from STR profiles (http://www.hprg.com/hapest5/index.html). The paternal haplotype for each sample was designated by its full 19-STR locus profile.

Y chromosome lineages (haplogroups) were defined as the unique combinations of SNP and STR data present in the samples. DYS389b was calculated by subtracting DYS389I from DYS389II, which was used for all statistical network analyses. Each male sample was assigned a SNP haplogroup following the conventions outlined by the Y-chromosome Consortium^93,184^ and detailed in PhylotreeY^188^. All of the Y-STR haplotypes were further checked for their haplogroup status using Athey’s (http://www.hprg.com/hapest5/) and the Nevgen Y-DNA (http://www.nevgen.org/) haplogroup predictors. The SNPs and STR alleles defined the haplogroups and haplotypes, respectively, for each male individual.

#### Comparative populations

We compared the mtDNA and NRY data obtained from the five Pakistani ethnic groups to those from populations in South Asia, Central Asia, East Asia, Middle East and Europe in an effort to place the genetic histories of these five Pakistani ethnic groups within a broader framework. For the mtDNA analysis, we examined a total of 11,411 mtDNA HVS1 sequences, including 659 from this study and the rest from comparative populations in South, Central and East Asia, Europe, Caucasus, and the Near East (Table S9). In addition, we compared 12,519 Y-STR haplotypes including 678 from this study and the rest from comparative populations in South, Central and East Asia, Europe, Caucasus, and the Near East (Table S10). All Y-STR haplotypes were reduced to ten loci (DYS19, DYS389I, DYS398b, DYS390, DYS391, DYS392, DYS393, DYS437, DYS438, and DYS439) to allow for the broadest comparison possible.

#### Statistical analyses

Haplotype diversity (h), nucleotide diversity (p) and pairwise differences were calculated for mtDNA HVS-1 sequences (np 16,024–16,400) and Y-STR haplotypes using Arlequin v. 3.5.2.1^189^. Descriptive statistical indices, as well as Tajima’s D^190^ and Fu’s FS^191^ neutrality tests, were calculated using the same software. Pairwise F_ST_ and R_ST_ distances values between populations were calculated from HVS-1 sequences and Y-STR haplotypes using the same software. F_ST_ values were estimated with the Tamura and Nei (1993) model of sequence evolution. The resulting matrices were visualized in Neighbor-joining trees using MEGA version X^192^. For both the mtDNA and NRY data sets, the genetic structure of Pakistani and comparative populations was examined through analysis of molecular variance (AMOVA) in Arlequin v. 3.5.2.1^189^.

#### Phylogenetic analysis

Median-joining networks^193^ were constructed for both mtDNA HVS-1 sequences and Y-STR haplotypes using Network version 5.0.1.1^193,194^ to explore the phylogenetic history of the genetic lineages encompassed within the five sampled ethnic groups. For mtDNA HVS-1 sequences, the mutation-weighting scheme was based on that described by Bandelt and coworkers^195^, in which fast-evolving sites were given lower weights relative to less mutable sites. All variants known to result from homopolymeric C expansions (e.g., A16182C, A16183C) or to occur at mutational hotspots in the mtDNA CR (e.g., T16519C) were excluded from the haplotypes used in this analysis.

The NRY haplotypes used to generate the networks for specific haplogroups consisted of 17 Y-STR loci. Y-STR loci were weighted according to their individual mutation rates^196^ by applying a fivefold weighting scheme with higher weights given to slowly evolving markers and lower weights to faster evolving markers. The multicopy marker DYS385 was not used in the analysis because the differentiation between its alleles was not possible to ascertain using the Y-Filer kit^187^.

### Geospatial frequency maps

The GPS coordinates for the KPP and comparative populations were determined and used to create geospatial maps of haplogroup frequencies with QGIS Desktop v.3.20.0 using the EPSG 4326 coordinate system. The resulting maps were exported at a scale of 1:48,000,000. Continent and country boundary vector data were procured from free-use, publicly available World Health Organization assets. The data points were organized by latitude/longitude coordinates from Tables S3 and S7, with the geospatial coordinates being used as the sample points in an Inverse Distant Weighted (IDW) interpolation calculation. A weight of 3 was used in these calculations to help clarify the produced raster visualization’s color ramp and decrease the known disadvantage of IDWs with irregular sample point distributions that produces visual peaks and pits around sample points. The resulting rasters were then clipped by the vector land boundaries and their color ramps clipped to 20 values between the min and max before being exported in PDF format^197,198^. This interpolation does not take into account natural land boundaries, water boundaries, or cultural boundaries that would affect the falloff of influence from neighboring sample points, since it is solely based on geographic distance.

## Supplementary Information

Supplementary Information 1.

Supplementary Information 2.

## Data Availability

The majority of the data discussed in this paper are provided in the Supplementary Tables, including mtDNA control region sequences and Y-SNP and Y-STR data. The mtDNA HVS1 sequences have also been deposited in the NCBI GenBank at https://www.ncbi.nlm.nih.gov/genbank/ under Accession numbers XXXXX-XXXXX.
