# Supplementary material for: Contrasting maternal and paternal genetic histories among five ethnic groups from Khyber Pakhtunkhwa, Pakistan
Source: Sci Rep. 2022 Jan 19;12:1027. doi: 10.1038/s41598-022-05076-3 (PMC8770644; doi:10.1038/s41598-022-05076-3)
Supplement: Supplementary file 1 — Supplementary Information 1. [file 41598_2022_5076_MOESM1_ESM.docx]

**Supporting Information**

**Table S1.** mtDNA haplogroup frequency distributions in KPP ethnic groups

**Table S2:** mtDNA haplotypes in KPP populations

**Table S3:** mtDNA haplogroup frequencies in KPP and comparative populations

**Table S4.** Pairwise F*_ST_* estimates based on mtDNA HVS1 sequences for all populations included in the regional comparative analyses. The pairwise F*_ST_* values appear below the diagonal and the corresponding p-values above the diagonal.

**Table S5.** Frequency of NRY haplogroups in the five KPP ethnic groups

**Table S6.** Y-STR data of the five ethnic groups

**Table S7:** NRY haplogroup frequencies in KPP and comparative populations.

**Table S8.** Pairwise R*_ST_* distances based on 10 Y-STR haplotypes from the five KPP ethnic groups and comparative populations. The pairwise R*_ST_* values appear below the diagonal and the corresponding p-values above the diagonal.

**Table S9.** Populations examined and their sample sizes.

**Table S10.** Populations examined and their sample sizes.

**Supplementary Text**

**Populations**

***Gujars*.** The Gujars are a Gujari-speaking (a lowland Indic-Aryan language) ethnic group, whose traditional occupation was nomadic herding. They inhabit the northwestern region of the Indian subcontinent spanning across the region of Jammu and Kashmir, Himachal Pradesh, Rajasthan, and Haryana and in Gujrat in India, as well as live in Pakistan and Afghanistan. Gujars represent one of the numerically largest ethnic groups in Pakistan whose approximately 2.3 million members reside primarily within the Punjab Province, largely in less agriculturally desirable mountainous areas. Concerning the origin of the Gujars, one hypothesis asserts that they originated in Central Asia and then, in the 3^rd^ century CE, migrated to India along with Huns and established themselves in Rajasthan ^1^. They subsequently migrated from Rajasthan to the Punjab, and further north into Kashmir and Himachal Pradesh Punjab, and then expanded into KPP during the 16^th^ century CE. Proponents of an alternative hypothesis ^2-5^ claim that the Gujars came to India from their homeland in Georgian Caucasus via Afghanistan in the 5^th^ century CE. These two hypotheses yield very different scenarios for the arrival of the Gujars in KPP. Under the former, Gujars represent relatively recent immigrants whose KPP residence encompasses only a half millennium and is of equal antiquity to that proposed for Pathan-speaking groups from Afghanistan ^6^. In the latter, Gujars represent long-standing residents of KPP (*c.* 1500 years) and originated among populations from the Caucasus.

***Jadoons*.** According to Weston ^7^, Jadoons are a Pashto-speaking (Eastern Iranian language) group that originally lived on the western slopes of the White Mountain range and the Nangarhar Province of Afghanistan. They subsequently migrated westward into the Kabul Valley during the 16^th^ century CE. In the 16^th^ century, they joined the Yousafzais and migrated eastwards to the Peshawar area. Once there, they settled in areas previously inhabited by the Dilazak, another immigrant ethnic group of Afghans, and eventually relocated along the western bank of the Indus River in a region now found within the Swabi District ^7^.

***Syeds*.** The Syeds are an ethnic group whose members claim descent from the Prophet Muhammad through his grandsons Hassan and Hussein, who were the sons of Hazrat Fatima, the youngest daughter of the Prophet, who lived 1,400 years ago ^8-10^. They are found throughout the Muslim world ^11^. It is commonly maintained that the Syeds migrated many centuries ago from different parts of the Middle East and Central Asia (Turkestan), including during the invasion of the Mongols and other periods of turmoil, such as the Ghaznavid dynasty, the Delhi Sultanate, and the Mughal Empire, which encompass a time span extending from the 15^th^ to the late 19^th^ century CE. Once present in the region, they migrated to the Sindh, Uch and Attock regions (Punjab Pakistan) in the north, and to the KPP, where they lived for several hundred years.

***Tanolis*.** Tanolis are speakers of Hindko, a term encompassing a number of western Punjabi dialects spoken in several discontinuous areas of northwestern Pakistan, with theirs being recognized as distinctive ^12^. This language is mutually intelligible with Punjabi and Saraiki ^13^, but shows closer affinities with the former than with the latter ^14^. The Tanolis mostly reside within the Tanawal Valley of KPP, although some families may be found in Swabi District and in Afghanistan. There is debate as to whether the Tanolis are actually Afghan Pashtuns, since they are sometimes viewed as being a Barlas Turkic group related to the Mongols that adopted many Pashtun cultural features ^15^.

The earliest written records indicate that Tanolis were settled in the present Trans-Indus region of Tanawal in Hazara prior to the Yousafzais invasion of KPP in the mid-15^th^ century. At the beginning of the 17^th^ century, the tribal territory of the Tanolis was invaded and temporarily occupied by Yousafzais under the leadership of Sultan Ali Asghar ^16^. Following this invasion, the Turk Rajas of Pakhli Sarkar, who were powerful vassals of the Mughal Empire, extended their authority over the Tanolis. In 1644, under the leadership of Akhund Salak Kabalgrami, the Tanoli chiefs Mimara Khan Palal and Chara Khan Hindwal drove the Turk Rajas out of their tribal territories. They established the state of Amb in Hazara and ruled until the wars with Sikhs in the 19^th^ century. They frequently engaged in rebellions against successive rulers of the Delhi Sultanate as well as served as allies to Ahmad Shah Abdali in his conquest of India. Because of their martial spirit, Charles Allen (2012: 96) referred to them as “the extremely and powerful Tanolis of the Tanawal Mountain.”

***Yousafzais*.** Yousafzais are the numerically largest Pashto-speaking (Eastern Iranian branch, Indo-European) ethnic group of KPP. They are further subdivided into a number of subtribes known as *khels* or *zais*  ^6,17-21^. According to Yousafzai oral traditions, their ancestral home was located in Kandahar in southern Afghanistan. Later, they moved eastward by way of Kabul, crossing the Khyber Pass into present day KPP near Peshawar ^6^. By the mid-15^th^ century CE, the Yousafzais began to move into the Swat Valley, displacing the Dilazaks (another Pathan ethnic group from Afghanistan) residing there ^17^.

**Supplementary Figures**

***Phylogenetic Analysis***

Specific mtDNA haplogroups stood out as being important for elucidating the genetic relationships among the five sampled ethnic groups. They derive from one of the three major macrohaplogroups defining all non-African maternal lineages, namely M, N and R (e.g., Quintana-Murci et al.^22^). Median-joining (MJ) networks for the derivative haplogroups were generated from HVS1 haplotypes present in the five ethnic groups to explore these relationships.

The network for macrohaplogroup M was characterized by the presence of a number of high frequency nodes, with long branches extending from them (**Supplementary Fig. 1a**). The majority represented South Asian haplogroups, with East Asian haplogroups such as M9 also being present. Because of the ubiquity of M-derived haplogroups in South Asian populations, the larger nodes often contained haplotypes from multiple ethnic groups. The network for mtDNA haplogroups C and D showed a similar pattern of haplotype diversity and sharing (**Supplementary Fig. 1b**).

The network for haplogroup N showed the main derived haplogroups in KPP populations, with long branches extending from them (**Supplementary Fig. 1c**). The central node for the commonly observed haplogroup W contained mtDNAs from four of the five KPP ethnic groups, the exception being Yousafzais, with other haplotypes from this lineage being present in all of the populations.

Many haplogroups deriving from macrohaplogroup R were present in the five KPP ethnic groups. Because of this fact and considering the large number of haplotypes for each haplogroup in these populations, we analyzed subsets of them through MJ network analysis, as described below.

The haplogroup HV network consisted of one large central node and several high frequency nodes (**Supplementary Fig. 1d**). The central node was defined by the revised Cambridge Reference Sequence (rCRS), which belongs to haplogroup H2 ^23^, with individuals from all five KPP ethnic groups having this haplotype. Two other large nodes for HV and H haplotypes also contained mtDNAs from four of the five samples.

The network for haplogroup U was composed of its many subbranches (U, U1, U2, U3, U4, U5, U6 and U7) (**Supplementary Fig. 1e**). Several high frequency nodes that were shared by two or more populations were observed in the network (U2a, U2b, U2e, U4), with the largest node representing the founder haplotype for U7 (#284). As noted above, this haplotype was found at moderate frequency among individuals from all five ethnic groups. Interesting, U7 likely spread into South Asia prior to the Holocene (~11.5 kya) and before it was dispersed into West Eurasia several thousand years later ^24^.

The macrohaplogroup R network for the KPP ethnic groups was comprised of 12 different haplogroups (**Supplementary Fig. 1f**). The most extensive was haplogroup T, which appeared in all five ethnic groups, with subbranches T1 and T2 clearly being defined in the network. The second major cluster was haplogroup R5, which also contained mtDNAs from all five ethnic groups. The third largest cluster was J1b, although only one haplotype (#172; J1b) was shared among the different ethnic groups. In addition, haplogroup K (which is actually a subbranch of U) also formed a separate branch in the network. Its single major node (#186; K1a) was the only one shared by multiple ethnic groups. Other less frequently occurring haplogroups (e.g., R2, R6, R30) appeared as smaller branches.

**Supplementary Figure 1**

Median-joining networks based on the HVS-1 haplotypes present in the five KPP populations. (a) Haplogroup M; (b) Haplogroups C and D; (c) Haplogroup N*; (d) Haplogroups H and HV; (e) Haplogroup U*; and (f) Haplogroup R*. Mutations occurring between np 16024-16400 are indicated along the branches.

**Figure S1a**


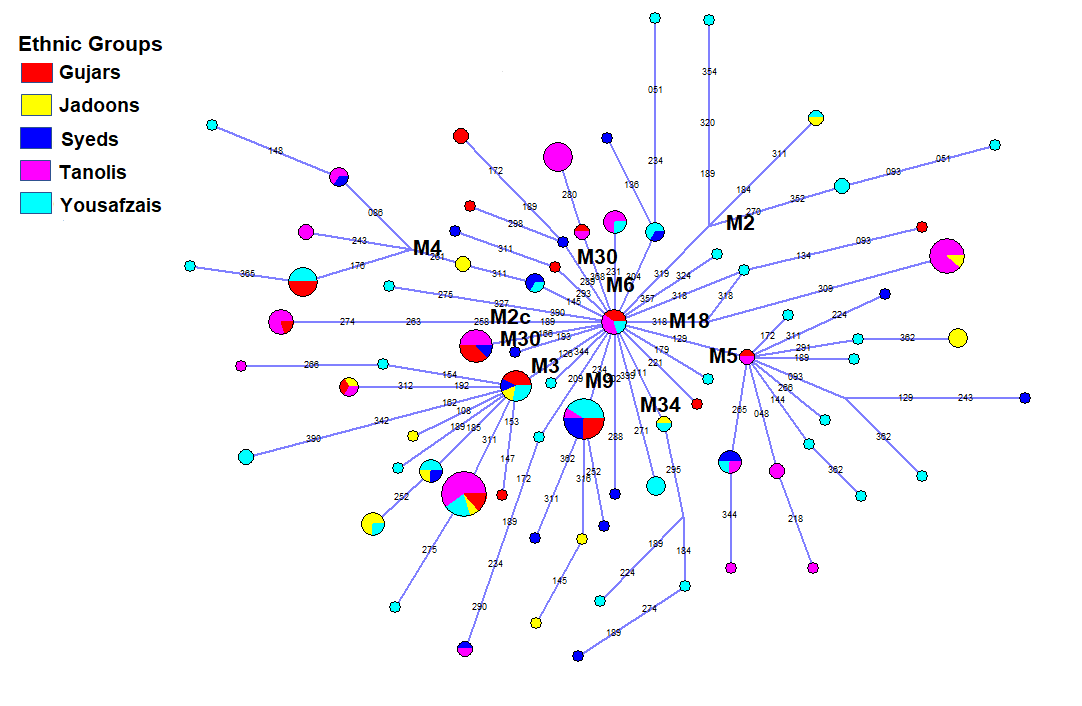


**Figure S1b**


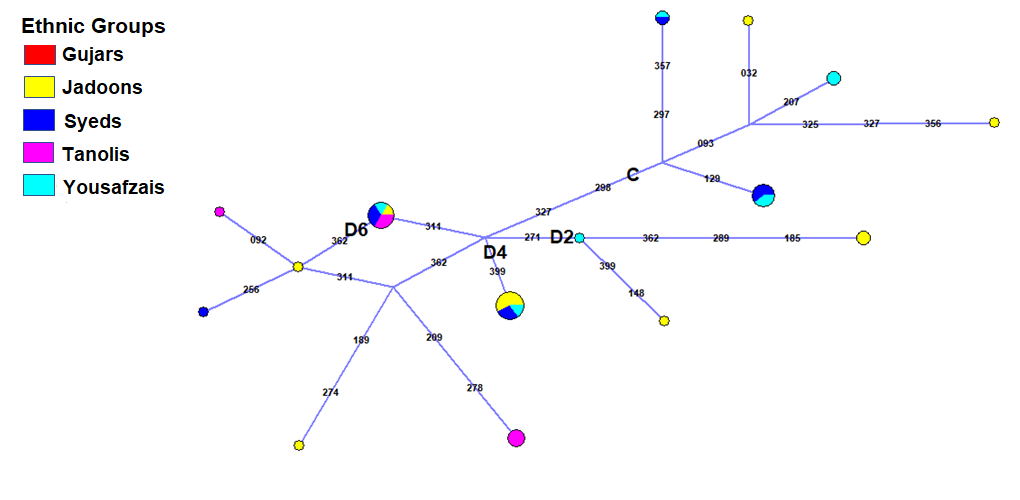


**Figure S1c**


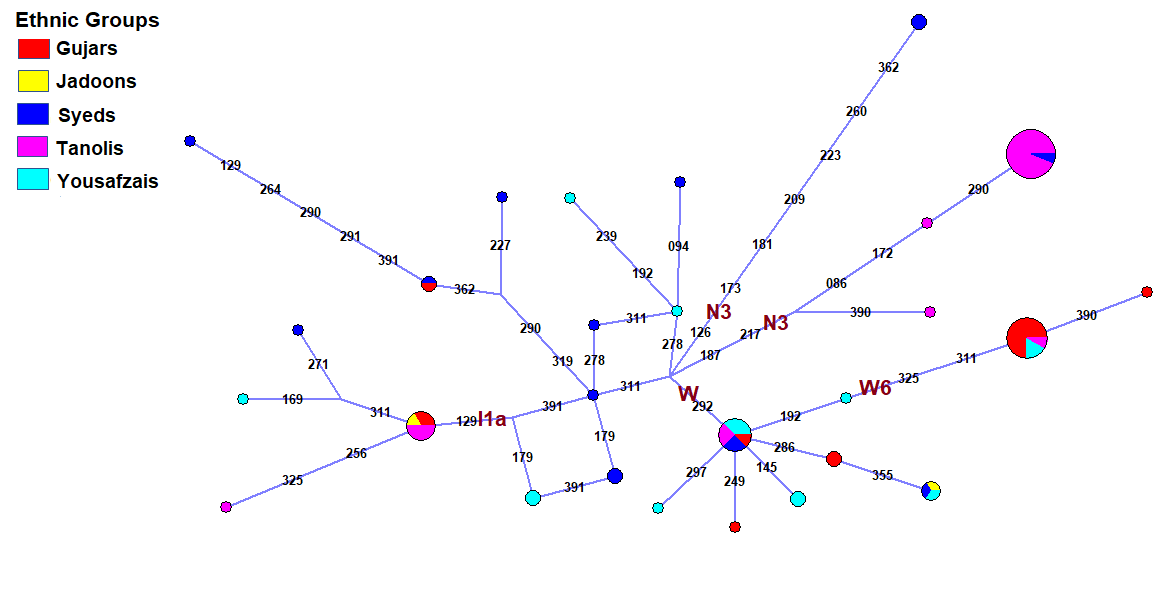


**Figure S1d**


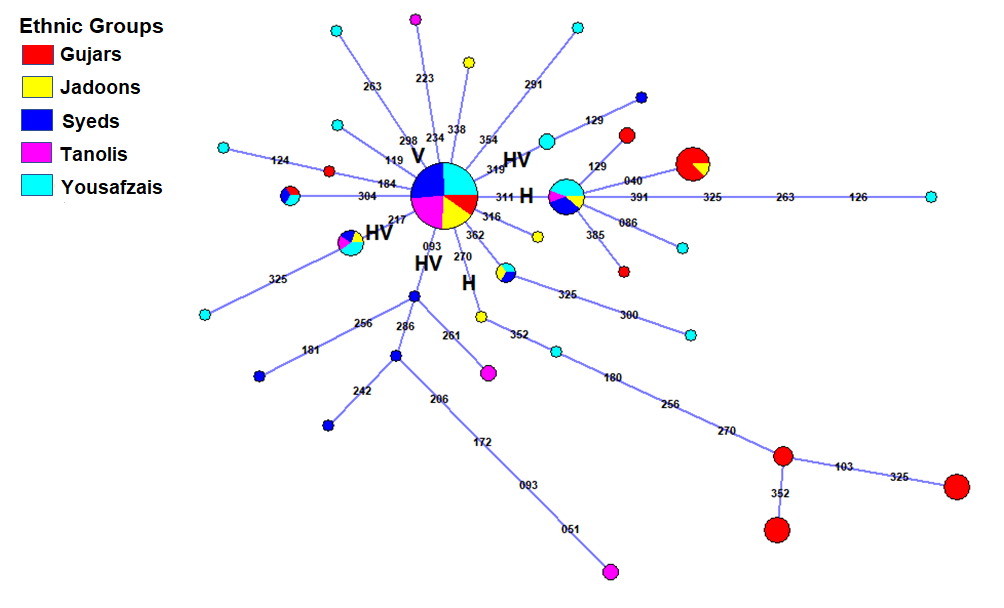


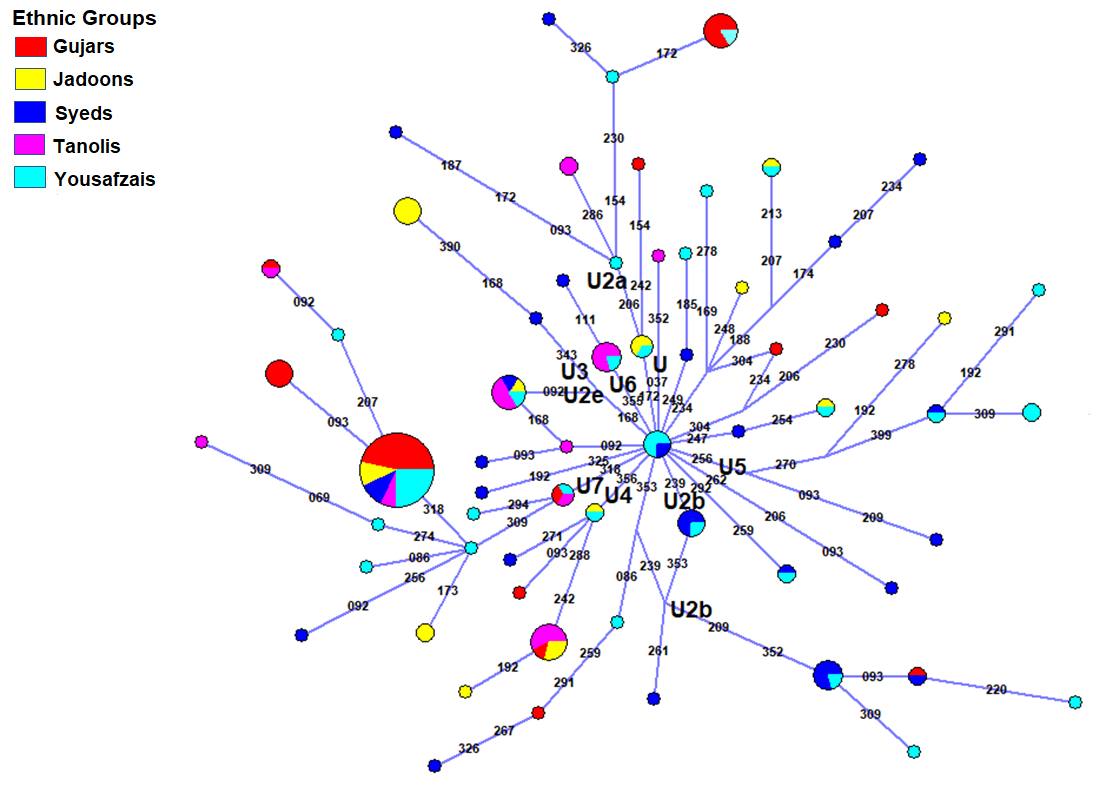
**Figure S1e**


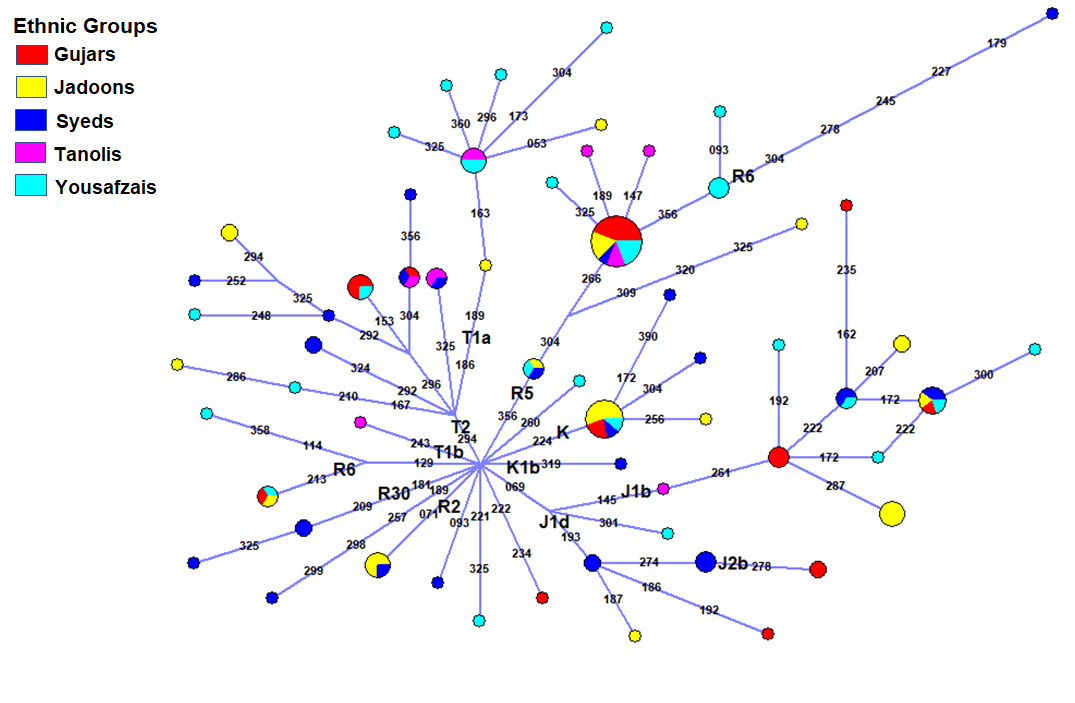
**Figure S1f**

***Phylogenetic Analysis***

The analysis of NRY diversity in KPP populations yielded a total of 295 distinct Y-STR haplotypes in these groups based on data for 19 loci (**Table S6**). Using Y-STR haplotypes, we constructed MJ networks for the four most common haplogroups (R1a1a-M17, R1b1-M297, O3-M122 and L-M20) within these populations.

*Haplogroup R1a1a-M17***.** We detected a total of 123 distinct Y-STR haplotypes among the 339 Y-chromosomes belonging to this lineage. The five ethnic groups shared few haplotypes, although certain haplotypes appeared at moderate frequencies within them. Four large nodes were present in this MJ network, with each largely comprised of haplotypes from Gujars, Syeds and Yousafzais, respectively, with the remaining haplotypes appearing on smaller branches scattered around the larger nodes (**Supplementary Fig. 2a**). Overall, the network exhibited a number of reticulations, most likely due to small number of mutational differences between the Y-STR haplotypes.

*Haplogroup R1b-M269.* A total of 26 distinct haplotypes were defined in 118 samples belonging to this paternal lineage. Nearly all of these haplotypes appeared in Tanolis, with the exception of four haplotypes shared with Jadoons, Syeds and Yousafzais (**Supplementary Fig. 2b**). The latter four may reflect admixture between Tanolis and neighboring populations.

*Haplogroup O3*. Thirty-one distinct haplotypes were delineated in 94 samples belonging to this paternal lineage. Jadoon haplotypes formed the core of the MJ network for this haplogroup, with one Yousafzai haplotype also appearing with them (**Supplementary Fig. 2c**). Otherwise, the O3 haplotypes in Gujars, Tanolis and Yousafzais appeared on long branches extending well away from the Jadoon haplotypes, suggesting they belonged to different subbranches of this lineage, hence, were likely acquired through interactions with other populations.

*Haplogroup L.* We identified a total of 24 distinct haplotypes for the 48 samples belonging to this paternal lineage. In the MJ network of these haplotypes, there was no clear pattern of distribution in the network, although the haplotypes for the Gujars and Syeds were generally separated from those in Jadoons and Yousafzais (**Supplementary Fig. 2d**).

**Supplementary Figure 2**

Median-joining networks based on 17 Y-STR haplotypes in the five KPP populations. (a) Haplogroup R1a; (b) Haplogroup R1b; (c) Haplogroup O3; and (d) Haplogroup L. In these networks, each node represents the relative frequency of a particular Y-STR haplotype, with its representation in each population indicated by different colors.

**Figure S2a**


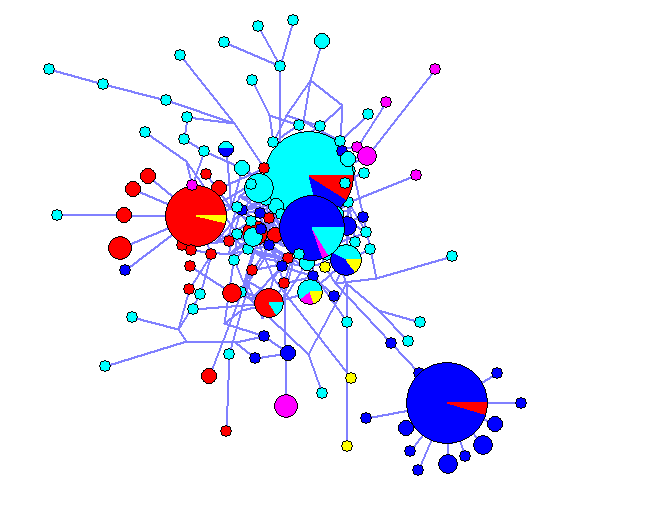

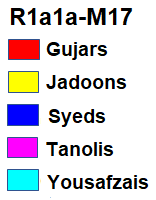


**Supplementary Figure 2b**


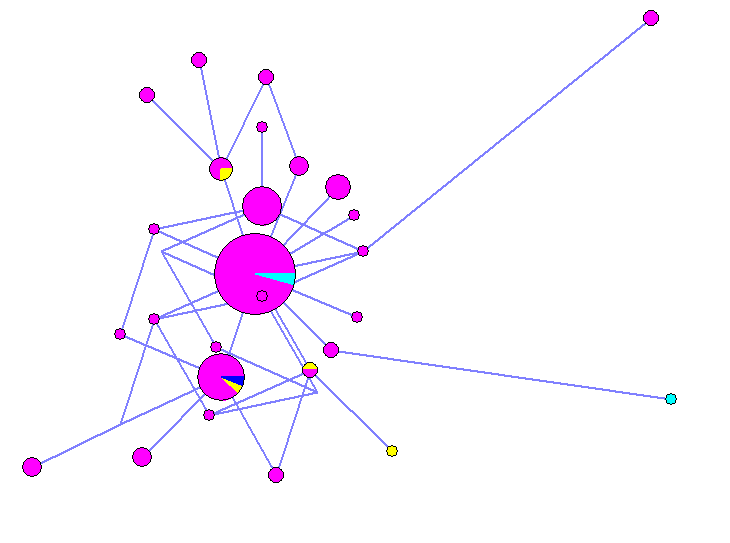

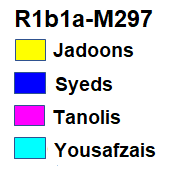


**Supplementary Figure 2c**


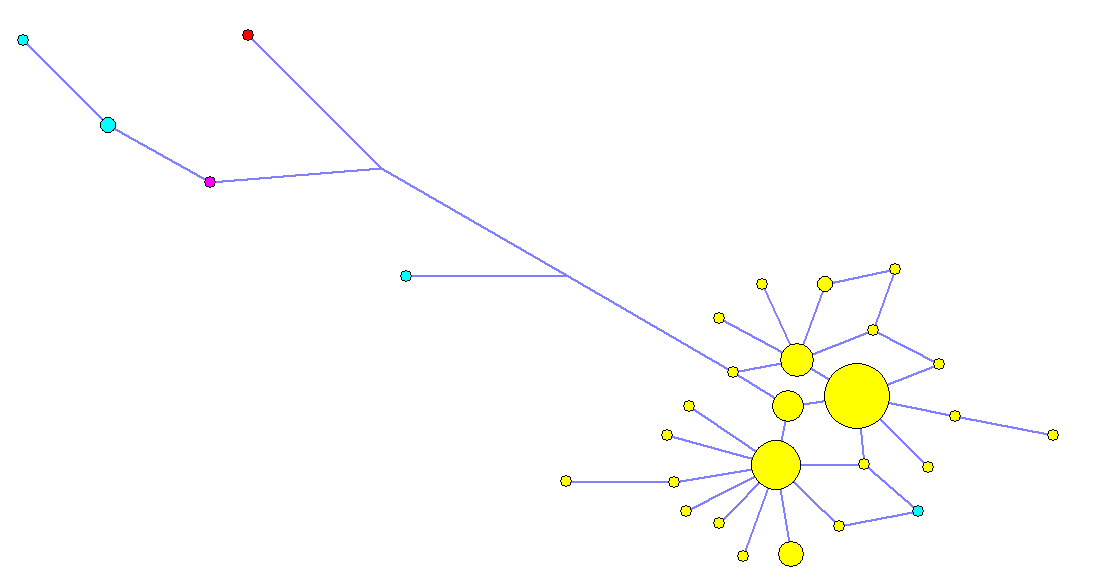

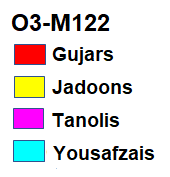


**Supplementary Figure 2d**


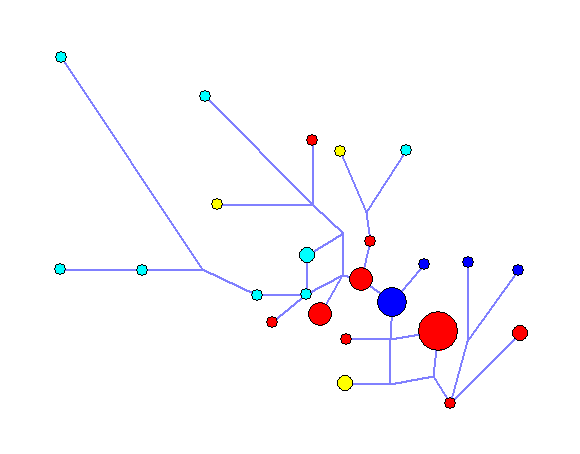

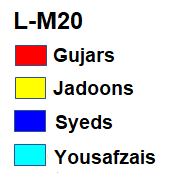


**References**

1 Wikely, J. M. Punjabi Mussalman. *Baltimore Penguin press.* (1985).

2 Tyagi, V. P. *Martial races of undivided India*. (Gyan Publishing House, 2009).

3 Bose, S. *Kashmir: Roots of conflict, paths to peace*. (Harvard University Press, 2009).

4 Qamar, R. *et al.* Y-chromosomal DNA variation in Pakistan. *Am J Hum Genet* **70**, 1107-1124, doi:10.1086/339929 (2002).

5 Nidvi, M. S. A. H. Sihrat Syed Ahmad Shaheed. H. M. . *Saeed and Company, Karachi, Pakistan* (1985).

6 Caroe, O. The Pathans. *Oxford University Press, London* (1958).

7 Weston, S. T. Glossary of the tribes and castes of the Punjab and N. W. F. Province. *Reprinted at the CIvil and Mililitary Gazette Press. Lahore.* (1911).

8 Ho, E. The graves of Tarim genealogy and mobility across the Indian Ocean. *Berkeley: University of California Press.* (2006).

9 Belle, E. M., Shah, S., Parfitt, T. & Thomas, M. G. Y chromosomes of self-identified Syeds from the Indian subcontinent show evidence of elevated Arab ancestry but not of a recent common patrilineal origin. *Archaeological and Anthropological Sciences* **2** (2010).

10 Ahmad, Z. Muslim caste in Uttar Pradesh. *The Economic Weekly* **14**, 325-336. (1962).

11 Levy, R. The social structure of Islam. Second edition of the sociology of Islam. *Cambridge University Press, Cambridge* (1957).

12 Bailey, T. G. *Languages of the Northern Himalayas: Being Studies in the Grammar of Twenty-Six Himalayan Dialects*. (Cambridge University Press, 2013).

13 Rahman, T. & Knight, T. *Language and politics in Pakistan*. (Oxford University Press Karachi, 1996).

14 Shackle, C. Problems of classification in Pakistan Panjab. *Transactions of the Philological Society* **77**, 191-210 (1979).

15 Watson, H. D. Gazetteer of Hazara District. *London: Chatto and Windus* (1907).

16 Bellew, H. W. *A general report on the Yusufzais*. (Sang-e-Meel Publications, 1864).

17 Barth, F. Political Leadership among Swat Pathans *The Athlone Press, London* (1959).

18 Nusser, M. & Dickore, W. B. A tangle in the triangle: vegetation map of the eastern Hindukush (Chitral, northern Pakistan). *Erdkunde* **56**, 37-59 (2002).

19 Khan, T. M. The Tribal Areas of Pakistan, a Contemporary Profile. *Lahore: Sang-e-Meel Publications.* (2008).

20 Coningham, R. & Young, R. The Archaeology of South Asia: From the Indus to Asoka, c. 6500 BCE–200 CE Cambridge. *Cambridge University Press* (2015).

21 Böhner, J. & Lucarini, V. Prevailing climatic trends and runoff response from Hindukush–Karakoram–Himalaya, upper Indus Basin. *Earth System Dynamics* **8** (2017).

22 Quintana-Murci, L. *et al.* Genetic evidence of an early exit of Homo sapiens sapiens from Africa through eastern Africa. *Nat Genet* **23**, 437-441, doi:10.1038/70550 (1999).

23 Finnilä, S., Lehtonen, M. S. & Majamaa, K. Phylogenetic network for European mtDNA. *Am J Hum Genet* **68**, 1475-1484, doi:10.1086/320591 (2001).

24 Sahakyan, H. *et al.* Origin and spread of human mitochondrial DNA haplogroup U7. *Sci Rep* **7**, 46044, doi:10.1038/srep46044 (2017).
